# Cognitive aging outcomes are related to both tau pathology and maintenance of cingulate cortex structure

**DOI:** 10.1002/alz.14515

**Published:** 2025-01-14

**Authors:** Stefania Pezzoli, Joseph Giorgio, Xi Chen, Tyler J. Ward, Theresa M. Harrison, William J. Jagust

**Affiliations:** ^1^ Department of Neuroscience University of California Berkeley California USA; ^2^ Molecular Biophysics and Integrated Bioimaging Lawrence Berkeley National Laboratory Berkeley California USA; ^3^ School of Psychological Sciences, College of Engineering Science and the Environment University of Newcastle Newcastle New South Wales Australia; ^4^ Department of Psychology Stony Brook University Stony Brook New York USA

**Keywords:** Alzheimer's disease, brain atrophy, brain maintenance, cognitive decline, exceptional cognitive performance, PET, successful aging, superaging, tau

## Abstract

**INTRODUCTION:**

Successful cognitive aging is related to both maintaining brain structure and avoiding Alzheimer's disease (AD) pathology, but how these factors interplay is unclear.

**METHODS:**

A total of 109 cognitively normal older adults (70+ years old) underwent amyloid beta (Aβ) and tau positron emission tomography (PET) imaging, structural magnetic resonance imaging (MRI), and cognitive testing. Cognitive aging was quantified using the cognitive age gap (CAG), subtracting chronological age from predicted cognitive age.

**RESULTS:**

Lower CAG (younger cognitive age) was related to slower decline in episodic memory, multi‐domain cognition, and atrophy of the midcingulate cortex (MCC). Lower entorhinal cortical tau was linked to slower decline in episodic memory, multi‐domain cognition, and hippocampal atrophy.

**DISCUSSION:**

These results suggest that aging outcomes may be influenced by two independent pathways: one associated with tau accumulation, affecting primarily memory and hippocampal atrophy, and another involving tau‐independent structural preservation of the MCC, benefiting multi‐domain cognition over time.

**Highlights:**

- Younger cognitive age (lower cognitive age gap [CAG]) is related to slower cognitive decline.
- Lower CAG is linked to slower midcingulate cortex (MCC) atrophy.
- Reduced tau in the entorhinal cortex is related to less hippocampal atrophy and cognitive decline.
- Structural preservation of the MCC benefits multi‐domain cognition over time.
- Two independent pathways influence cognitive aging: tau accumulation and MCC preservation.

## 1 BACKGROUND

Variability in cognitive performance increases with advancing age, with substantial heterogeneity in cognitive aging trajectories. Some individuals experience cognitive decline associated with brain pathologies and age‐related processes, whereas others show preserved or even exceptional cognitive functioning.^1^ Various studies have aimed to elucidate the neural features linked to exceptional cognitive performance in older adults. These individuals can be referred to as successful cognitive agers (SAs), and have been described previously using different terms including SuperAgers,^2^, ^3^, ^4^ supernormals,^5^, ^6^ superior,^7^ or optimal^8^ memory performers.

**FIGURE 1:**
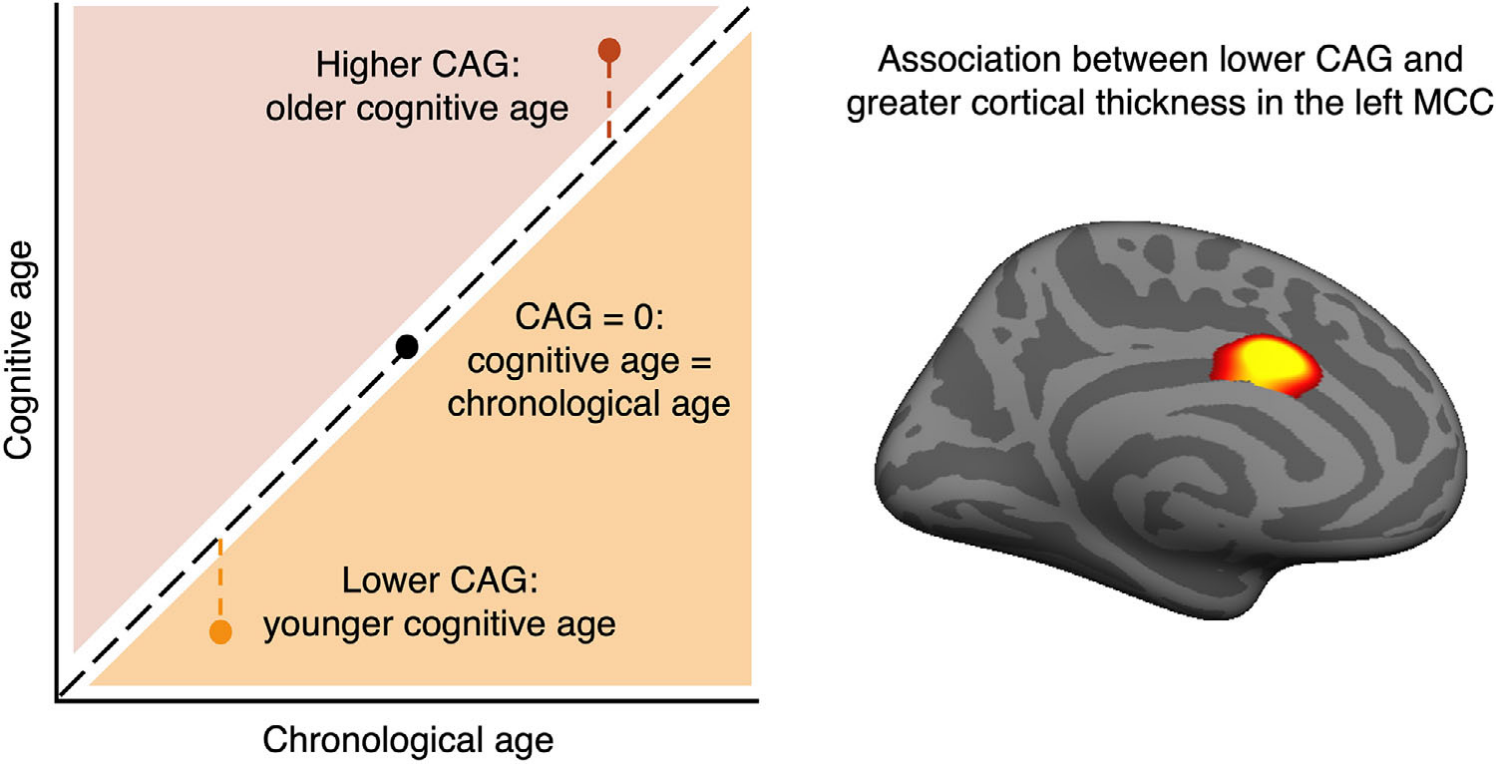
Lower CAG is associated with thicker MCC. On the left, an illustration depicting CAG scores computed by subtracting the chronological age from the corresponding cognitive age, where lower scores indicate younger cognitive age. On the right, previous results from vertex‐wise analyses revealing a significant negative association between CAG scores and MCC thickness (adapted from Pezzoli et al.^9^). CAG, cognitive age gap; MCC, midcingulate cortex.

Prior research has used a number of strategies to define SAs, often based on selecting those with exceptional performance based on thresholds^5^, ^8^, ^9^ or comparison to younger individuals.^2^, ^4^, ^7^ In a recent study from our laboratory,^9^ we defined SAs by using neuropsychological tests spanning multiple cognitive domains to estimate predicted age, and then defining a cognitive age gap (CAG) by subtracting the chronological age from their corresponding cognitive age. Notably, we observed a robust relationship between lower CAG, indicating better cognitive performance than expected for age, and thicker midcingulate cortex (MCC)^9^ (Figure 1). This same brain region is notable; previous reports using a variety of definitions of SAs reported greater cortical thickness^2^, ^10^ and higher metabolism^11^ there. In addition to findings in MCC, individuals with younger cognitive age also showed larger hippocampal volumes and less deposition of pathological tau aggregates in the entorhinal cortex (EC).^9^ In this context, the CAG can be conceptualized as a continuous measure of cognitive aging, particularly sensitive to brain features related to SA. However, it is uncertain whether having a younger cognitive age also denotes slower cognitive decline over time in older individuals.

Although investigating the differences between SAs and typical agers is valid to understand the neural features associated with normal and superior cognitive performance, here we took a different approach. We used continuous CAG scores to measure the continuum of cognitively normal aging, ranging from exceptional to typical cognitive functioning. Previous studies relied predominantly on cross‐sectional data. Although there is limited evidence indicating slower atrophy over time in the total cortical volume and total gray matter (GM) volume in SA, there are conflicting results regarding rates of hippocampal atrophy.^3^, ^12^, ^13^ There are no studies specifically addressing whether SAs show less longitudinal structural atrophy in the anterior cingulate cortex (ACC)/MCC, or whether SAs experience less longitudinal tau accumulation. These questions have profound implications for our understanding of cognitive aging. It is unclear whether SAs show a slower cognitive decline due to less age‐associated pathology, or whether they are the beneficiaries of early life advantages that persist throughout their lifespan.

**FIGURE 2:**
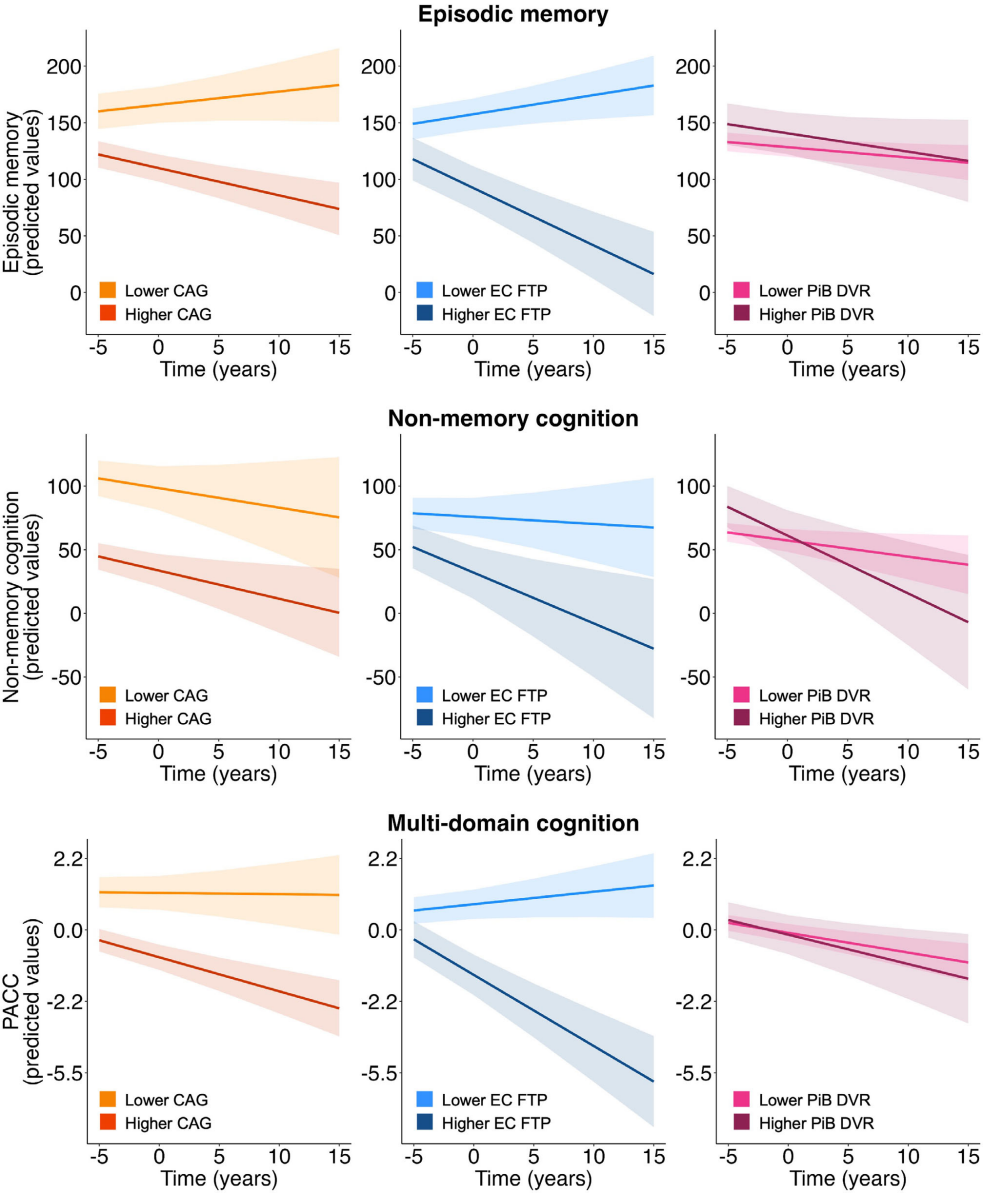
Effects of cross‐sectional CAG, EC tau, and Aβ on longitudinal change in EM, non‐memory, and multi‐domain cognition composite scores. CAG, EC FTP SUVR, and PiB DVR were included in the same linear mixed‐effects model for each composite score, controlling for sex and years of education. Predicted composite scores are plotted over time, measured in years. Lower CAG and EC FTP SUVR were significantly associated with slower decline in EM and multi‐domain cognition. Lower PiB DVR was significantly associated with slower decline in NM. Continuous predictors were mean‐centered. CAG, EC FTP SUVR, and PiB DVR were included as a continuous variable in the models but represented as mean ± 1 SD for visualization purposes. CAG, cognitive age gap; DVR, distribution volume ratio; EC, entorhinal cortex; EM, episodic memory; FTP, 18F‐Flortaucipir; NM, non‐memory cognition; PACC, Preclinical Alzheimer Cognitive Composite; PiB, 11C‐Pittsburgh compound B; SUVR, standardized uptake value ratio.

**FIGURE 3:**
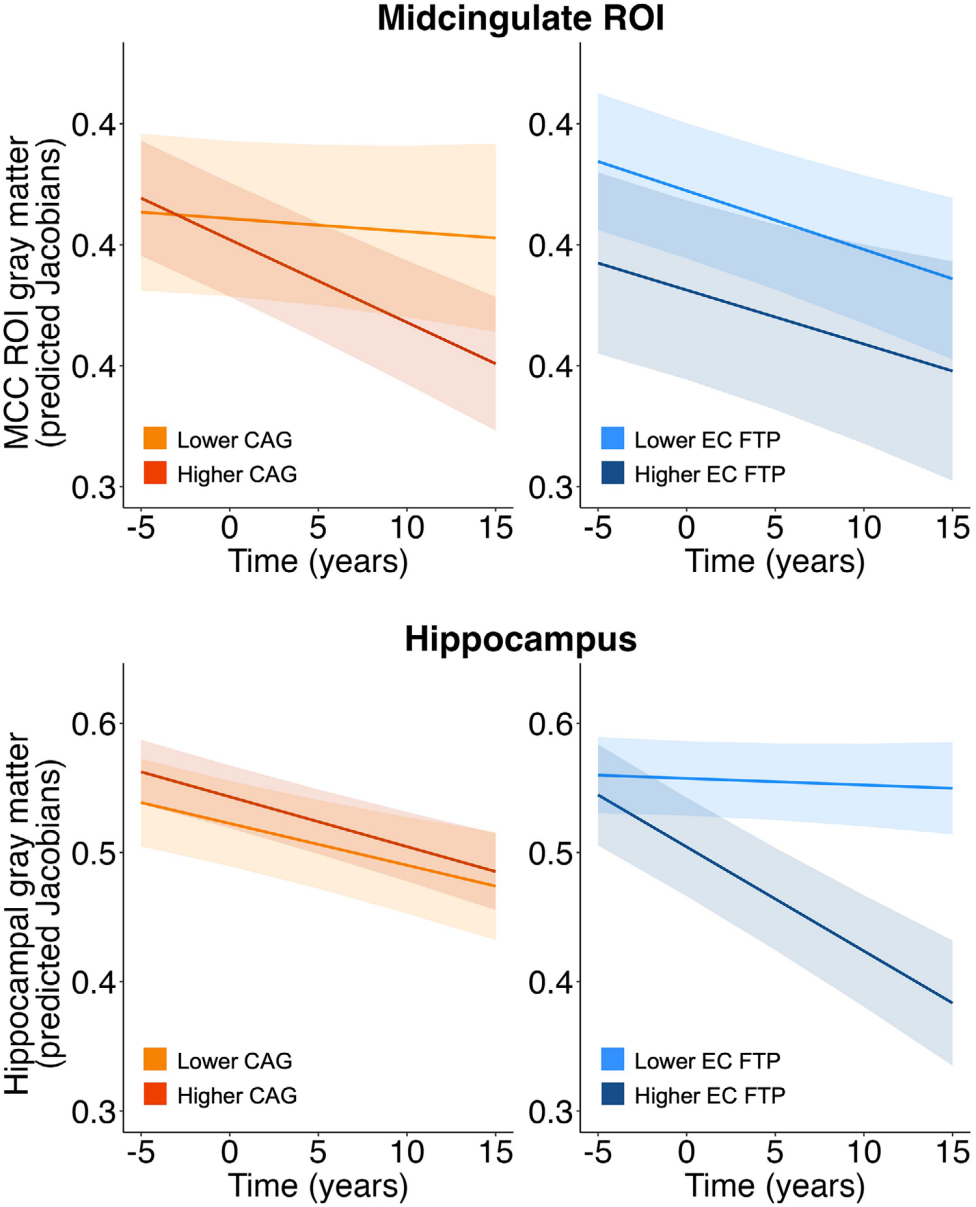
Effects of cross‐sectional CAG and EC tau on longitudinal GM change in a MCC ROI and hippocampus. CAG, EC FTP SUVR, PiB DVR, sex, and years of education were included in the same linear mixed‐effects model for each GM ROI. Predicted Jacobians are plotted over time. Lower CAG was associated with slower atrophy progression in the MCC ROI. Continuous predictors were mean‐centered. CAG and EC FTP SUVR were included as a continuous variable in the models but represented as mean ± 1 SD for visualization purposes. CAG, cognitive age gap; DVR, distribution volume ratio; EC, entorhinal cortex; FTP, 18F‐Flortaucipir; GM, gray matter; MCC, midcingulate cortex; PiB, 11C‐Pittsburgh compound B; ROI, region of interest; SUVR, standardized uptake value ratio.

Our goal was to examine relationships between cross‐sectional CAG and longitudinal changes in (1) cognition (results described in Section 3.2 and displayed in Figure 2), (2) brain structure (results described in Section 3.3 and displayed in Figure 3), and (3) positron emission tomography (PET)–measured tau pathology (results described in Section 3.4). Finally, we investigated whether (4) change in cognition was associated with change in MCC GM, EC tau, and amyloid beta (Aβ) burden (results described in Section 3.5). In more detail, we explored longitudinal changes in composite scores for episodic memory (EM) and non‐memory cognition (NM)^14^ and in the multi‐domain Preclinical Alzheimer Cognitive Composite (PACC).^15^ Changes in GM structure were investigated in the hippocampus and in a region of interest (ROI) using a mask in the MCC region defined from our previous study, where we found greater cortical thickness associated with younger cognitive age (MCC ROI; Figure 1).^9^ We explored relationships between CAG scores and changes in each cognitive composite and GM, and investigated whether and how PET‐measured Aβ and tau affected these relationships. Finally, we examined whether tau accumulation in the EC and inferior temporal (IT) cortex, and global Aβ accumulation varied based on CAG scores. We hypothesized that lower CAG, indicating younger cognitive age, would be associated with better longitudinal outcomes.

## 2 METHODS

### 2.1 Sample

This study involved 109 community‐dwelling cognitively normal older adults from the Berkeley Aging Cohort Study (BACS). Because cognition is significantly affected by increasing age, age is a crucial factor to incorporate in defining SAs.^16^ Consequently, the present study included only participants 70 years of age or older, in line with prior research on SAs.^5^, ^9^, ^13^ Inclusion criteria involved a baseline Mini‐Mental State Examination (MMSE) score ≥25, normal daily functioning, and no history of neurological disease or major medical illness affecting cognition, history of substance abuse, or depression. Participants remained cognitively unimpaired throughout the study. Other demographic and clinical features available included the following: years of education, apolipoprotein E (*APOE*) genotyping, history of hypertension, self‐reported family history of dementia, and Geriatric Depression Scale (GDS) scores. All participants included in the present investigation underwent two or more cognitive testing sessions, with a minimum of one tau‐PET imaging scan with ^18^F‐Flortaucipir (FTP) and one Aβ‐PET session using ^11^C‐Pittsburgh compound B (PiB). A subgroup had two or more T1 structural magnetic resonance imaging (MRI) scans and PiB scans (*n* = 92), and a subgroup underwent two or more FTP PET scans (*n* = 72).

We aimed to investigate the impact of Alzheimer's disease (AD) pathology on the association between cross‐sectional CAG (cognitive‐age – chronological age) and (1) cognitive decline, and (2) longitudinal GM atrophy. For this reason, for each participant, we focused on cross‐sectional CAG scores derived from the cognitive sessions that closely matched FTP scans (and corresponding PiB). For 83% of participants (*n* = 91), the time between FTP scan and CAG‐related cognitive assessment was less than 6 months, and less than a year for 94% (*n* = 103); for six participants, time interval was slightly over a year (1.15 ± 0.13 years). Because FTP PET was introduced later in the BACS protocol, to optimize the use of all cognitive sessions available for each participant, our longitudinal analyses incorporated cognitive sessions conducted after the age of 70 both before and after the cross‐sectional CAG for most participants. Notably, 96% (*n* = 105) of the participants had at least one prospective neuropsychological evaluation, whereas only four participants had only retrospective cognitive assessments available. Similarly, to maximize the use of all MRI scans available for each participant, our longitudinal analyses incorporated MRI scans undertaken after the age of 70 both before and after the cross‐sectional CAG aligned with the FTP scans; 82% (*n* = 75) of the participants had at least one prospective structural MRI scan. This approach allowed us to gain a more comprehensive understanding of participants' cognitive and structural changes over time, particularly focusing on longitudinal trajectories after the age of 70.

The institutional review board at the University of California, Berkeley, and the Lawrence Berkeley National Laboratory (LBNL) reviewed and approved the study, and all participants provided written, informed consent.

**RESEARCH IN CONTEXT**

1. **Systematic review**: We conducted a comprehensive literature search using PubMed to explore longitudinal brain features linked to successful cognitive agers (SAs. Previous studies primarily used cross‐sectional data, and the limited longitudinal evidence showed conflicting results regarding the rate of hippocampal atrophy in SA. There is a lack of research addressing longitudinal structural changes in the midcingulate cortex (MCC) and tau accumulation in SA.
2. **Interpretation**: Our findings suggest that aging outcomes may be influenced by independent pathways. One is associated with tau accumulation, affecting primarily memory decline and hippocampal atrophy, and the other appears to involve tau‐independent structural preservation of the MCC with beneficial effects on multi‐domain cognition over time.
3. **Future directions**: Future research is needed to explore the specific mechanisms by which tau‐independent preservation of the MCC contributes to successful cognitive aging. Further work may focus on understanding how these findings relate to genetic and modifiable risk factors.

### 2.2 Neuropsychological assessment

The BACS protocol includes a comprehensive neuropsychological assessment evaluating a variety of cognitive domains such as verbal and visual memory, working memory, processing speed, executive functioning, and attention. In the present study, the following tests were used: the California Verbal Learning Test (CVLT), Logical Memory, Visual Reproduction, Trail Making Test (TMT) A and B, Stroop test, digit symbol task, phonemic verbal fluency F‐A‐S test, Animal Naming, Vegetable Naming, and digit span forward and backward. Repeated cognitive assessment was available for all participants. EM and NM composite scores were calculated using confirmatory factor analyses (CFA) in BACS, as described in detail previously.^14^ The EM composite was computed using the following tests: CVLT Short Delay Free Recall (SDFR), CVLT Long Delay Free Recall (LDFR), Visual Reproduction I, Visual Reproduction II, Logical Memory Total Score, and Verbal Paired Associates. The NM composite included the following tests: Stroop in 60 s, Digit Symbol, TMT‐A, TMT‐A subtracted from TMT‐B (Trails B–A), Digit Span Backward, Animal Naming, and Vegetable Naming. In the present study, multi‐domain cognition was measured with the PACC, a composite that was designed to be sensitive to early AD‐related cognitive changes.^15^, ^17^ In the present study, the PACC was calculated using the MMSE, Logical Memory total recall, Digit Symbol, and the CVLT LDFR.^15^ To compute the PACC, raw individual cognitive scores were *z*‐transformed within the sample using baseline cognitive assessment's mean and standard deviation (SD). Then, *z*‐scores for each cognitive test were summed at each time point, and the resulting sum was standardized to the mean and SD of the baseline sum score.^15^

### 2.3 Cognitive age model

Partial least squares regression (PLSr) was used to estimate participants’ cognitive‐predicted age from cognitive tests, as described in detail previously.^9^ The cognitive age model was developed using independent data sets for training (*n* = 293; 55–96 years of age at baseline) and testing (*n* = 238; 56–97 years of age at baseline), with chronological age serving as the response variable. The training and test cohorts did not differ in age, years of education, history of hypertension, diabetes, or heart disease, but the training set had a greater proportion of female participants.^9^ The model was built using all available neuropsychological sessions from the training cohort (*n* = 457 sessions), and the learned parameters were subsequently applied to predict cognitive age in the test cohort (*n* = 1141 sessions). Predictors encompassed various tests and subtests (*n* = 19), contributing to a comprehensive evaluation of cognitive functioning. These included measures for EM, executive functioning, processing speed, and language skills. The following tests/subtests were included: CVLT Trials 1‐5 Free Recall total, CVLT SDFR, CVLT Short‐Delay Cued Recall (SDCR), CVLT LDFR, CVLT Long‐Delay Cued Recall (LDCR), TMT‐A and B, Stroop in 60 s, FAS test, Animal Naming, Vegetable Naming, Digit Symbol, Logical Memory total recall, Visual Reproduction I, II and recognition total, Digit Span Forward and Backward, and Boston Naming Test. To address potential practice effects, session number was also included as a predictor, bringing the total number of predictors to 20. The trained model accurately predicted age in the independent test cohort with a mean absolute error of 4.36. The variance explained was similar in the test and training samples, reaching 41% in the test sample and 49% in the training set.^9^ A statistical bias correction was then applied to each predicted age to adjust for a commonly observed bias in age prediction models, where younger adults' ages tend to be overestimated, and older adults' ages underestimated. We used a method based on the correction proposed by Beheshti et al.,^18^ which has been utilized previously in brain age prediction models. CAG (cognitive‐age – chronological age) was then computed for each session to represent individual variability in cognitive aging, where lower values indicate better cognitive performance than expected for age (younger cognitive age, indicating successful aging).^9^ In this study, CAG scores reflect age‐bias‐corrected CAG scores, computed by subtracting chronological age from the bias‐corrected cognitive age values. The sample is a subset of participants selected from the test cohort in our previous study.^9^

### 2.4 Image acquisition

All participants underwent at least one tau‐PET imaging visit using FTP and at least one Aβ‐PET imaging visit using PiB, which were performed on a BIOGRAPH PET/CT scanner with previously described protocols.^19^, ^20^, ^21^ A subset of 92 participants had a minimum of two PiB scans, and 72 participants underwent a minimum of two FTP scans. FTP and PiB were synthesized at the LBNL Biomedical Isotope Facility. CT scans acquired prior to each PET scan were used for attenuation correction. For FTP scans, participants were injected with 10 mCi of tracer and scanned from 80 to 100 min post‐injection. For PiB scans, 90 min of dynamic emission data frames were acquired post‐injection of 15 mCi of tracer. FTP and PiB images were reconstructed using an ordered subset expectation maximization algorithm with scatter correction and smoothed with a 4‐mm Gaussian kernel.

A subgroup of 92 participants underwent a minimum of two high‐resolution T1‐weighted magnetization‐prepared rapid gradient echo (MPRAGE) structural MRI scans acquired on a 1.5 T Siemens Magnetom Avanto scanner at LBNL. The following acquisition parameters were applied: repetition time (TR) = 2110 ms, echo time (TE) = 3.58 ms, flip angle = 15°, 1 mm slice thickness, and 1 × 1 mm^2^ in‐plane resolution.

### 2.5 Image processing

FTP standardized uptake value ratio (SUVR) images were created using the mean tracer uptake 80–100 min post‐injection and normalized to the inferior cerebellar GM reference region.^22^ To account for partial volume effects, we used Geometric transfer matrix partial volume correction (PVC) on the Desikan–Killiany FreeSurfer‐derived ROIs for FTP data processing.^23^, ^24^ The Desikan–Killiany atlas was used to define ROIs of the EC as an early region of tau deposition and the IT cortex as an early‐stage tau accumulation region outside the medial temporal lobe.^25^, ^26^, ^27^, ^28^ Models including EC and IT FTP were first run using PVC data, and then rerun using non‐PVC data. For models involving MCC ROI FTP, we used non‐PVC SUVRs. When exploring longitudinal changes in tau, FTP scans were normalized using white matter reference regions. In particular, a cerebral white matter reference region was used for PVC data, and an eroded subcortical white matter reference region for non‐PVC data.^29^ This approach was chosen due to previous evidence reporting more stable estimates of FTP change over time.^29^, ^30^

To calculate PiB distribution volume ratio (DVR), we used Logan graphical analysis on PiB frames over 35‐90 min post‐injection, with normalization using a reference region in the cerebellar GM.^31^, ^32^ We computed global cortical PiB DVR using FreeSurfer‐derived cortical ROIs.^33^, ^34^ A global PiB DVR threshold of 1.065 was used to determine Aβ positivity.^19^ To calculate Centiloid (CL) values we used a conversion equation previously used in our laboratory and developed for our processing pipeline: CL = (DVR x 142.73) – 141.99.^9^, ^35^

Structural MRI processing was performed using Statistical Parametric Mapping 12 (SPM12; (https://www.fil.ion.ucl.ac.uk/spm/) longitudinal registration^36^ and DARTEL (Diffeomorphic Anatomical Registration using Exponentiated Lie algebra)^37^ implemented in MATLAB R2023a (MathWorks Inc., www.mathworks.com/). Specifically, we used the serial longitudinal registration to create (1) one midpoint, average structural MRI image for each participant; and (2) one Jacobian map for each MRI scan timepoint, representing the deformation of each scan to the midpoint average. Then each midpoint image was segmented into GM, white matter, and cerebrospinal fluid (CSF) in native space. Next, GM‐segmented midpoint images were used to create GM‐weighted Jacobian maps at each timepoint. The DARTEL toolbox was employed to generate a study‐specific template and to warp individual GM‐weighted Jacobian images into Montreal Neurological Institute (MNI) space. Smoothing was applied to the images using a full width at half maximum (FWHM) 8 mm Gaussian kernel. This procedure resulted in a smoothed, normalized GM‐weighed Jacobian map at each timepoint for each participant.

To examine regional GM change in regions associated with SAs, we first created an ROI mask in the MCC. This mask was based on prior vertex‐wise analyses, revealing an association between lower CAG, indicating a cognitive age younger than expected for one's chronological age, and greater cortical thickness.^9^ The cortical surface resulting from the vertex‐wise analyses was clustered with a threshold of *p* ≤ 0.0001 and subsequently transformed to MNI space. The resulting surface label was used to create a cortical volume ROI, referred to as the MCC ROI, created using Freesurfer 7.1.0 (http://freesurfer.net/). An ROI encompassing the left and right hippocampus was obtained using the Brainnetome atlas (https://atlas.brainnetome.org/). The mean voxel values for the selected ROIs were extracted from each GM‐weighted Jacobian image using the MarsBaRtoolbox toolbox for SPM (http://marsbar.sourceforge.net/).

### 2.6 Statistical analyses

Statistical analyses were performed using R version 4.3.1 (https://www.r‐project.org/). We ran linear mixed‐effects models (LMEMs) using the lme4 R package to investigate the effects of cross‐sectional CAG and AD pathology on longitudinal changes in EM, NM, and PACC composite scores across all available cognitive sessions after the age of 70. In addition, we explored the impact of CAG and AD pathology on longitudinal atrophy in GM ROI (MCC ROI and bilateral hippocampus) across all structural MRI scans after the age of 70. In separate models, each cognitive composite and GM ROI was included as the outcome variable. Time, cross‐sectional CAG, cross‐sectional FTP SUVR, and cross‐sectional PiB DVR were included as predictor variables, with CAG × time, FTP SUVR × time, and PiB DVR × time as our main predictors of interest. Sex and years of education were included as covariates, as well as each covariate by time interactions. Random effects included subject intercept and time slope. Continuous predictors and covariates were mean‐centered. For each composite score and GM ROI (Jacobians), the following model was performed: Cognitive composite/GM ROI ∼ time + CAG * time + EC FTP SUVR * time + PiB DVR * time + sex * time + education * time + random slope + random intercept. All models were repeated using IT FTP SUVR as the tau pathology measure. First, each model was conducted using FTP PVC SUVRs. Subsequently, to assess any potential influence of PVC data, we repeated the models using non‐PVC data. The analyses were also repeated using the time between the CAG cognitive session and the FTP PET scan as a covariate of no interest. Finally, we restricted the analyses to participants with an interval between the CAG cognitive session and FTP PET of less than 6‐months.

In another set of LMEMs, we aimed to investigate the effects of cross‐sectional CAG on longitudinal tau. In separate models, the outcome variables were EC and IT PVC FTP SUVRs, whereas CAG, CAG × time, PiB DVR, PiB DVR × time were included as predictors. Sex, sex × time, years of education, and years of education × time were included as covariates of no interest. Subject intercept and time slope were included as random effects. Continuous independent variables were mean‐centered. The following models were performed: EC or IT FTP SUVR ∼ time + CAG * time + PiB DVR * time + sex * time + education * time + random slope + random intercept. All models were repeated using non‐PVC FTP SUVRs. We also explored longitudinal change FTP MCC ROI SUVR, and whether it varied by cross‐sectional CAG and PiB DVR.

In a subgroup of participants with a minimum of two cognitive sessions, FTP, PiB and MRI scans (*n* = 72), we used LMEMs to estimate longitudinal slopes for EM, NM, PACC, regional MCC GM atrophy, EC FTP SUVR, and global PiB DVR. We used Pearson partial correlations and multiple linear regression models to assess the relationships between EM, NM, and PACC longitudinal slopes and (1) MCC GM atrophy, (2) FTP, and (3) PiB slopes. Age, sex, and years of education were included as covariates. The following multiple regression model was performed for EM, NM, and PACC: Cognitive composite slope ∼ MCC ROI GM Jacobian slope + EC FTP SUVR slope + PiB DVR slope + age + sex + years of education.

To control for multiple comparisons, we applied false discovery rate (FDR) correction specifically to the interaction terms with time, our primary predictors of interest (CAG × time, ERC SUVR × time, IT SUVR × time, and PiB DVR × time where applicable). Corrections were applied separately within each analysis set (cognitive composites, GM ROIs, PET measures). In addition, FDR correction was applied across three regression models using cognitive slopes as outcome measures.

## 3 RESULTS

### 3.1 Cohort characteristics

Cohort characteristics at the cognitive sessions closest to FTP scans after the age of 70 are summarized in Table 1. The mean (± SD) age of participants was 77.47 ± 5.04 with 58% females and a mean of 16.97 ± 1.85 years of education. Within our study cohort, 45% were Aβ positive, determined using a PiB DVR threshold of 1.065^19^; 25% were *APOE* ε4 carriers (ε3/ε4), 8% were *APOE* ε2 (ε2/ε3), and 66% were *APOE* ε3/ε3 homozygotes. In addition, there were two ε2/ε4 carriers (no *APOE* ε2/ε2 and ε4/ε4 homozygotes). The average cognitive follow‐up time was 7.10 ± 3.77 years, with an average of 6.17 ± 3.28 cognitive sessions. In a subsample of 92 participants with two or more structural MRI scans, the mean follow‐up time was 5.91 ± 3.49 years with an average of 3.40 ± 1.28 scans. In a subset of 72 participants with at least two FTP scans, the average follow‐up time was 3.67 ± 1.98 years with an average of 2.65 ± 0.79 scans. Cohort characteristics for each subsample are reported in Table S1.

**TABLE 1:** Cohort characteristics (*n* = 109).

Characteristic	
Age	77.47 (5.04)
Sex, female, *n* (%)	63 (58)
Education, years	16.97 (1.85)
History of hypertension, years, *n* (%)	40 (37)
Family history of dementia, years, *n* (%)^a^	37 (35)
*APOE* ε4, *n* (%)^b^	26 (25)
PiB+, *n* (%)	49 (45)
PiB, CLs	23.43 (31.96)
CAG, years	−0.21 (4.69)
MMSE	28.69 (1.27)
GDS^c^	3.50 (3.10)

Race/ethnicity, n (%)	
Asian	5 (5)^d^
Black or African American	1 (1)
Native Hawaiian or other Pacific Islander	1 (1)
Hispanic or Latino	4 (4)^e^
White *n*	102 (94)
Cognition, years of follow‐up	7.10 (3.77)
MRI, years of follow‐up^f^	5.91 (3.49)
PiB, years of follow‐up^f^	5.92 (3.54)
FTP, years of follow‐up^g^	3.67 (1.98)

Abbreviations: CAG, cognitive age gap; CL, centiloid; FTP, ^18^F‐Flortaucipir; GDS, Geriatric Depression Scale; MMSE, Mini‐Mental State Examination; MRI, magnetic resonance imaging; PiB, ^11^C‐Pittsburgh compound B. Values represent mean (SD) and n (%) for continuous and categorical variables, respectively.

^a^ missing data for two participants.

^b^ missing data for four participants.

^c^ missing data for one participant.

^d^ Including Asian and Wpezzohite (*n* = 1).

^e^ Including Hispanic or Latino and White (*n* = 4) and Hispanic or Latino and Native Hawaiian or other Pacific Islander (*n* = 1).

^f^ *n* = 92.

^g^ *n* = 72.

### 3.2 Lower CAG predicts slower decline in EM and multi‐domain cognition, beyond the effects of Aβ and tau pathology

We used LMEMs to investigate longitudinal changes in EM and NM composite scores,^14^ and in the multi‐domain composite PACC.^15^ (See Figure S1 for a spaghetti plot displaying individual trajectories of cognitive scores over time.) We predicted that lower cross‐sectionally defined CAG, indicating younger cognitive age, should be related to better longitudinal cognitive outcomes, and investigated whether cross‐sectional AD pathology affected these relationships. The following model was examined separately for each composite score: cognitive composite ∼ time + CAG * time + EC FTP SUVR * time + PiB DVR * time + sex * time + education * time + random slope + random intercept.

In the model predicting EM decline, the two‐way interactions between time and CAG, and time and EC FTP SUVR, were significant (CAG × time: *β* = −0.15, *p* = 0.01, FDR *p* = 0.02; EC FTP SUVR × time: *β* = −4.87, *p* < 0.001, FDR *p* < 0.001), but not PiB DVR × time (*β* = −0.75, *p* = 0.52, FDR *p* = 0.52) (Figure 1). The full model is shown in Table 2. In the model predicting NM cognition decline, the PiB DVR × time interaction was significant, although not surviving correction for multiple comparisons (*β* = −3.41, *p* = 0.04, FDR *p* = 0.13), but not CAG × time (*β* = −0.03, *p* = 0.71, FDR *p* = 0.71) and EC FTP SUVR × time (*β* = −2.43, *p* = 0.11, FDR *p* = 0.11) (Figure 2 and Table 2). In the model predicting decline in the PACC, the two‐way interactions between time and CAG or EC FTP SUVR were significant (CAG × time: *β* = −0.005, *p* = 0.03, FDR *p* = 0.049; EC FTP SUVR × time: *β* = −0.21, *p* < 0.001, FDR *p* < 0.001), but not PiB DVR × time (*β* = −0.04, *p* = 0.47, FDR *p* = 0.52) (Figure 2 and Table 2). These results suggest that lower CAG scores and lower EC FTP burden were independently associated with a slower decline in EM and in the multi‐domain PACC, whereas lower PiB DVR was related to a slower decline in NM cognition. Using IT FTP SUVR as the measure of tau pathology, results were similar for both the EM model (IT FTP × time: *β* = −5.95, *p* = 0.003, FDR *p* = 0.004, CAG × time: *β* = −0.12, *p* = 0.046, FDR *p* = 0.14, PiB DVR × time: *β* = −1.16, *p* = 0.38, FDR *p* = 0.54) and the NM models (IT FTP × time: *β* = −1.36, *p* = 0.58, FDR *p* = 0.56, CAG × time: *β* = −0.03, *p* = 0.73, FDR *p* = 0.77, PiB DVR × time: *β* = −4.06, *p* = 0.02, FDR *p* = 0.06). In the model predicting PACC decline, IT FTP × time interaction was significant (*β* = −0.33, *p* < 0.001, FDR *p* < 0.001), but not CAG × time interaction (*β* = −0.003, *p* = 0.18, FDR *p* = 0.27). Full models are reported in Table S2. To ensure that these analyses were not influenced by the use of PVC of FTP data, we repeated the models using non‐PVC data, yielding comparable results. All results were similar when we added the number of days between the cognitive session (cross‐sectional CAG) and the FTP scan as a covariate of no interest. Furthermore, restricting the analyses to participants with less than a 6‐month interval between the CAG‐related cognitive session and the FTP scan yielded similar results.

**TABLE 2:** Predicting longitudinal change in cognition: LMEMs parameter estimates.

	Memory			Non‐memory			Multi‐domain cognition		
Parameter	Estimate	s.e.	*p*‐value	Estimate	s.e.	*p*‐value	Estimate	s.e.	*p*‐value
Intercept	130.68	3.27	<0.001	57.80	3.40	<0.001	−0.12	0.12	0.34
Time	−1.09	0.29	<0.001	−1.96	0.43	<0.001	−0.08	0.01	<0.001
CAG	−2.35	0.53	<0.001	−2.73	0.56	<0.001	−0.09	0.02	<0.001
EC FTP	−46.76	11.14	<0.001	−31.29	11.60	0.01	−1.78	0.41	<0.001
PiB DVR	12.89	12.19	0.29	4.18	12.69	0.74	−0.07	0.45	0.88
Sex	−0.17	5.04	0.97	−9.86	5.26	0.06	−0.41	0.19	0.03
Education	5.87	1.36	<0.001	5.09	1.42	<0.001	0.22	0.05	<0.001
CAG × time	−0.15	0.05	0.01^*^	−0.03	0.07	0.71^**^	−0.005	0.002	0.03^***^
EC FTP × time	−4.87	1.04	<0.001^****^	−2.43	1.49	0.11^*****^	−0.21	0.04	<0.001^******^
PiB DVR × time	−0.75	1.15	0.52^*******^	−3.41	1.62	0.04^********^	−0.04	0.05	0.47^*********^
Sex × time	0.60	0.46	0.20	0.06	0.67	0.93	0.02	0.02	0.31
Education × time	0.27	0.13	0.04	0.37	0.19	0.05	0.02	0.01	0.004

Abbreviations: CAG, cognitive age gap; DVR, distribution volume ratio; EC, entorhinal cortex; FTP, ^18^F‐Flortaucipir; LMEMs, linear mixed‐effects models; PiB, ^11^C‐Pittsburgh compound B; s.e., standard error.

* FDR *p* = 0.02.

** FDR *p* = 0.71.

*** FDR *p* = 0.049.

**** FDR *p* < 0.001.

***** FDR *p* = 0.11.

****** FDR *p* < 0.001.

******* FDR *p* = 0.52.

******** FDR *p* = 0.13.

********* FDR *p* = 0.52.

### 3.3 Lower CAG is associated with slower atrophy in the MCC, but not in the hippocampus

Structural MRI scans were processed using a longitudinal pipeline^36^ to generate GM‐weighted Jacobian maps. These maps represent the divergence of scans at each timepoint relative to a subject‐specific midpoint average image. We used LMEMs to investigate longitudinal GM atrophy in the MCC region that we previously found was associated with younger cognitive age defined at one timepoint (MCC ROI, Figure 1)^9^ and also in the hippocampus. The choice of these regions was informed by the previously reported associations between hippocampal and MCC structural integrity and SAs, defined as individuals exhibiting exceptional cognitive performance. Figure S2 shows spaghetti plots of GM Jacobian values over time across subjects, illustrating individual trajectories for the MCC ROI and hippocampus. We posited that lower CAG scores, indicating younger cognitive age, would be associated with slower atrophy progression. The following model was examined separately for the MCC ROI and the hippocampus: GM Jacobians ∼ time + CAG * time + EC FTP SUVR * time + PiB DVR * time + sex * time + education * time + random slope + random intercept.

In the model predicting MCC ROI GM atrophy, CAG × time interaction was significant (*β* = −0.0001, *p* = 0.007, FDR *p* = 0.01), but not EC × time (*β* = 0.0001, *p* = 0.88, FDR *p* = 0.88) (Figure 3 and Table 3). The results were similar using IT FTP SUVR as a measure of tau deposition (Table S3). Consistent results were found when years of education was removed from the models. Similar results were obtained when we restricted the analyses to participants with less than a 6‐month interval between the CAG‐related cognitive session and the FTP scan. In a model with only time as a predictor, we found a significant effect (*β* = −0.002, *p* < 0.001). In the model predicting hippocampal atrophy, EC × time was significant (*β* = −0.006, < 0.001, FDR *p* < 0.001), but not CAG × time (*β* = −0.00003, *p* = 0.62, FDR *p* = 0.62). When IT FTP SUVR was used as a measure of tau pathology, interactions for CAG × time (*β* = −0.00003, *p* = 0.59) and IT FTP SUVR × time (*β* = −0.003, *p* = 0.11) were not significant (Table S3). Consistent results were obtained when years of education was removed from the models. When we included only participants with less than a 6‐month period between the CAG‐related cognitive session and the FTP scan, IT FTP SUVR × time interaction was significant (*β* = −0.005, *p* = 0.02), but not CAG × time interaction (*β* = −0.00004, *p* = 0.55). Findings were replicated using non‐PVC data. In addition, we repeated the models including the MCC ROI FTP SUVR as the tau pathology measure. In the model predicting MCC ROI GM atrophy, CAG × time interaction was significant (*β* = −0.0001, *p* = 0.009), but not MCC ROI FTP × time (*β* = 0.0003, *p* = 0.68). In the model predicting hippocampal atrophy, interactions for CAG × time (*β* = −0.0001, *p* = 0.26) and MCC ROI FTP × time (*β* = −0.001, *p* = 0.36) were not significant. All results were similar when we included the number of days between the cognitive session (cross‐sectional CAG) and the FTP scan as a covariate of no interest.[Table alz14515-tbl-0003]

**TABLE 3 alz14515-tbl-0003:** Predicting longitudinal GM atrophy: LMEMs parameter estimates.

	MCC ROI			Hippocampus		
Parameter	Estimate	s.e.	*p*‐value	Estimate	s.e.	*p*‐value
**Intercept**	0.40	0.01	<0.001	0.54	0.01	<0.001
**Time**	−0.002	0.0002	<0.001	−0.004	0.0003	<0.001
**CAG**	−0.0003	0.001	0.73	0.001	0.0011	0.43
**EC FTP**	−0.02	0.02	0.18	−0.04	0.02	0.09
**PiB DVR**	−0.002	0.02	0.92	0.03	0.02	0.22
**Sex**	−0.01	0.01	0.15	−0.02	0.01	0.08
**Education**	0.001	0.002	0.54	−0.003	0.003	0.37
**CAG** × **time**	−0.0001	0.00003	0.007^*^	−0.00003	0.0001	0.62^**^
**EC FTP** × **time**	0.0001	0.0008	0.88^***^	−0.006	0.001	<0.001^****^
**PiB DVR** × **time**	−0.0009	0.0007	0.25^*****^	−0.00002	0.0011	0.98^******^
**Sex** × **time**	0.0005	0.0003	0.09	0.0001	0.0005	0.79
**Education** × **time**	−0.0002	0.0001	0.06	−0.00003	0.0001	0.81

Abbreviations: CAG, cognitive age gap; DVR, distribution volume ratio; EC, entorhinal cortex; FDR, false discovery rate; FTP, ^18^F‐Flortaucipir; GM, gray matter; LMEMs, linear mixed‐effects models; MCC, midcingulate cortex; PiB, ^11^C‐Pittsburgh compound B; ROI, region of interest; s.e., standard error.

* FDR *p* = 0.01.

** FDR *p* = 0.62.

*** FDR *p* = 0.88.

**** FDR *p* < 0.001.

***** FDR *p* = 0.50;.

****** FDR *p* = 0.98.

### 3.4 Tau and Aβ do not vary by CAG

LMEMs were used to explore longitudinal changes in tau within the EC as an early site of tau accumulation, as well as within the IT cortex as a region where tau deposition occurs at an early stage outside the medial temporal lobe (MTL).^25^, ^26^, ^27^, ^28^ The following models were examined to predict the longitudinal change in tau independently for EC, IT and MCC ROI: FTP SUVR ∼ time + CAG * time + PiB DVR * time + sex * time + education * time + random slope + random intercept. We used an LMEM to predict longitudinal change in PiB DVR: PiB DVR ∼ time + CAG * time + sex * time + education * time + random slope + random intercept.

In the model predicting EC tau accumulation, we found no significant PiB DVR × time (*β* = 0.04, *p* = 0.12, FDR *p* = 0.12) or CAG × time (*β* = −0.0003, *p* = 0.76, FDR *p* = 0.765) interactions. There was a significant main effect of PiB DVR (*β* = 0.31, *p* = 0.01, FDR *p* = 0.01). In the model predicting IT tau accumulation, the main effect of PiB DVR (*β* = 0.23, *p* = 0.001, FDR *p* = 0.001) and PiB DVR × time interaction were significant (*β* = 0.05, *p* = 0.02, FDR *p* = 0.048), but not CAG × time (*β* = −0.001, *p* = 0.34, FDR *p* = 0.69). Full models are reported in Table S4. These results suggest that tau accumulation rate did not vary based on CAG score. On the other hand, higher PiB DVR significantly predicted faster IT tau accumulation. Using non‐PVC data to predict EC tau accumulation, PiB DVR × time interaction was marginally significant (*β* = 0.03, *p* = 0.05), but not CAG × time (*β* = −0.0003, *p* = 0.67). There was a significant main effect of PiB DVR (*β* = 0.13, *p* = 0.03). Using non‐PVC data to predict IT tau accumulation, PiB DVR × time (*β* = 0.03, *p* = 0.06) and CAG × time (*β* = −0.0005, *p* = 0.45) interactions were not significant, but there was a significant main effect of PiB DVR (*β* = 0.10, *p* = 0.03). Finally, we examined longitudinal FTP uptake in the MCC ROI and found no significant CAG × time (*β* = 0.001, *p* = 0.41) and PiB DVR × time (*β* = 0.05, *p* = 0.25) interactions. There was, however, a significant main effect of PiB DVR (*β* = 0.29, *p* = 0.01), indicating that participants with higher levels of Aβ had higher tau deposition in the MCC ROI, irrespective of CAG. Nonetheless, no differences in the rate of change were observed. In a model with only time as a predictor, we found no significant effect (*β* = 0.01, *p* = 0.20), suggesting that tau in the MCC ROI does not significantly change over time. In the model predicting longitudinal change in PiB DVR, we found no significant CAG × time interaction (*β* = 0.0003, *p* = 0.49).

### 3.5 MCC atrophy is related to multi‐domain cognitive decline, beyond the effects of Aβ and tau pathology

LMEMs were used to estimate longitudinal slopes for EM, NM, PACC, regional GM atrophy, EC FTP SUVR, and global PiB DVR. Pearson partial correlations and multiple linear regression models were used to assess the relationships between the longitudinal slope of cognitive composites and (1) MCC GM atrophy, (2) FTP, and (3) PiB slopes, controlling for age, sex, and years of education. The following regression model was examined separately for each composite score: cognitive composite slope ∼ ROI GM Jacobian slope + EC FTP SUVR slope + PiB DVR slope + baseline age + sex + years of education.

**FIGURE 4:**
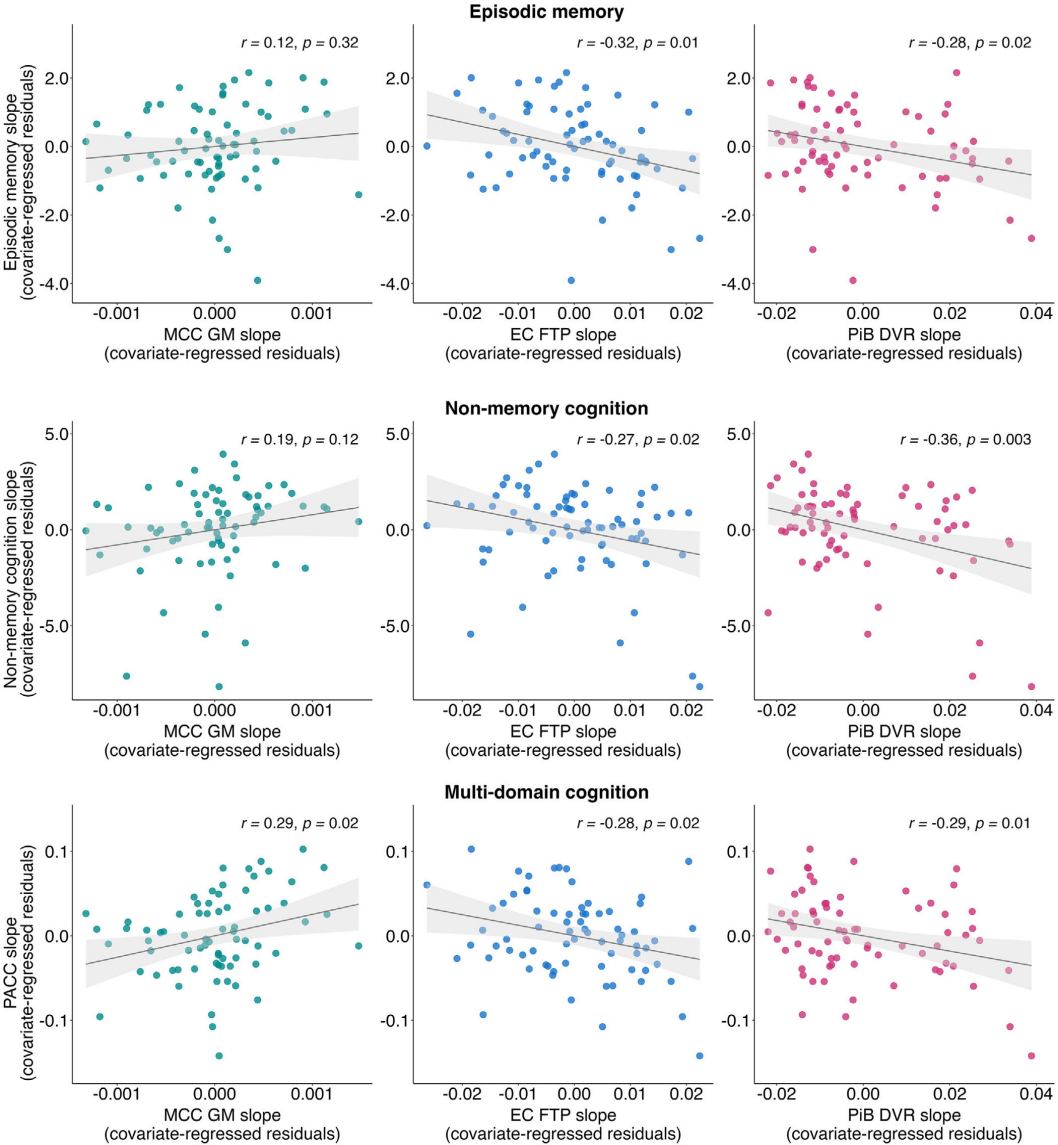
Linear relationships between each cognitive composite slope and (1) MCC GM Jacobian slope; (2) EC FTP slope; and (3) PiB DVR slope. Partial Pearson correlation coefficients (*r*‐values) and *p*‐values are reported, controlling for age, sex, and years of education. For visualization purposes, the plotted data points represent residuals from linear regression models, after adjusting for covariates. DVR, distribution volume ratio; EC, entorhinal cortex; FTP, 18F‐Flortaucipir; GM, gray matter; MCC, midcingulate cortex; PACC, Preclinical Alzheimer Cognitive Composite; PiB, 11C‐Pittsburgh compound B.

After adjusting for age, sex, and years of education, in separate partial correlations, EM slope showed significant associations with EC FTP slope (*r *= −0.32; *p* = 0.01) and PiB slope (*r* = −0.28; *p* = 0.02), but not with MCC GM slope (*r *= 0.12; *p* = 0.32) (Figure 4). When EC FTP slope, PiB slope, and MCC GM slope were included as predictors in the same regression model (adjusted *R* ^2^ = 0.11, *p* = 0.03), EC FTP slope was the only significant predictor of EM slope (standardized *β *= −0.26; *p* = 0.04, FDR *p* = 0.13), although not surviving correction for multiple comparisons. The full multiple regression model is reported in Table S5. Similarly, NM slope was associated with EC FTP slope (*r *= −0.27; *p* = 0.02) and PiB slope (*r* = −0.36; *p* = 0.003), but not with MCC GM slope (*r *= 0.19; *p* = 0.12) (Figure 4). In a multiple regression model (adjusted *R* ^2^ = 0.15, *p* = 0.01), PiB DVR slope was the only significant predictor of NM slope (standardized *β *= −0.32; *p* = 0.01, FDR *p* = 0.02) (Table S5). In contrast, PACC slope was associated with MCC GM slope (*r *= 0.29; *p *= 0.02) as well as EC FTP slope (*r *= −0.28; *p* = 0.02) and PiB slope (*r *= −0.29; *p* = 0.01). Multiple regression analyses (adjusted *R* ^2^ = 0.22, *p* = 0.001) revealed that both MCC GM slope (standardized *β *= 0.27; *p* = 0.02, FDR *p* = 0.058) and PiB slope (standardized *β *= −0.25; *p* = 0.02, FDR *p* = 0.04) were significant predictors of PACC slope (Table S5).

## 4 DISCUSSION

In this study, we quantified SAs using the CAG, which is a continuous measure of cognitive aging defined cross‐sectionally through a cognitive age model in older adults 70 years of age or older. We showed that participants with lower cross‐sectional CAG had slower cognitive decline in EM and PACC composite scores, as well as slower longitudinal MCC atrophy. Although these associations were not explained solely by the presence of AD pathology, tau in the EC was also related to memory and multi‐domain cognitive decline and to longitudinal hippocampal atrophy. Neither longitudinal hippocampal atrophy nor tau accumulation rates varied by CAG. We also found that slower multi‐domain cognitive decline was associated with slower MCC atrophy progression. On the other hand, EM decline was associated primarily with EC tau accumulation. Together, these results suggest separate and independent pathways affecting aging outcomes. One pathway involves tau accumulation, which affects hippocampal atrophy and memory. Another pathway is related to the preservation of MCC volume over time, which is independent of AD pathology and is associated with preserved multi‐domain cognition.

Our first aim was to investigate whether a lower cross‐sectional CAG was associated with slower cognitive decline, in addition to indicating a cognitive age younger than expected for one's chronological age. We found that lower CAG was associated with a slower decline in EM and the multi‐domain PACC. Notably, all the models also included measures of Aβ and tau. Predictably, higher levels of EC tau predicted a faster decline in both composite scores. These results suggest that CAG captures features of normal cognitive aging that are unaffected by Aβ and tau pathology. On the other hand, longitudinal decline in NM did not vary by CAG or tau; instead, it was associated with Aβ deposition. This result is consistent with previous findings that linked Aβ pathology to executive functioning and processing speed in cognitively normal older adults.^38^, ^39^, ^40^ This result, however, did not survive correction for multiple comparisons, and therefore needs to be interpreted with caution. It is not entirely clear why the CAG is more sensitive to longitudinal changes in EM and PACC, but not NM. The CAG includes multiple cognitive domains, including EM, executive functioning, processing speed, and language.

Cross‐sectional studies have repeatedly reported greater cortical thickness in ACC/MCC regions in SAs.^2^, ^9^, ^10^, ^41^ Although most findings pertain to individuals with exceptional performance in the memory domain, the criteria defining SAs varied across studies,^2^, ^7^, ^9^, ^10^ indicating the robustness of this result. Limited evidence indicates that SAs are characterized by slower global atrophy rates over time.^3^, ^12^ To the best of our knowledge, this is the first study showing differential SA‐related longitudinal GM changes in the MCC. Previous studies investigating longitudinal trajectories in brain structure in SA compared to typical agers found either no differences,^13^ or slower atrophy progression in other regions, including MTL areas.^12^ In this study, we found that adults 70 years of age or older with a younger cognitive age exhibited slower MCC atrophy progression. Critically, these neurobiological changes occurred independently of AD‐related processes. This result contributes to the existing literature on SAs by employing a novel methodology. It is important to note that we conceptualize cognitive aging on a continuum rather than relying on relatively arbitrary cutoffs and dichotomizations. Our finding further validates our approach and the use of CAG as a continuous measure of cognitive aging, particularly sensitive to SA‐related brain characteristics (e.g., MCC). Although previous results provide strong evidence of brain reserve, defined as the brain neurobiological status at a particular time point^42^ (i.e., greater ACC/MCC thickness), our study suggests that SAs may be characterized also by brain maintenance, specifically in the MCC (i.e., slower atrophy progression). In this context, better brain maintenance, as demonstrated by the relative preservation of brain morphology over time, could sustain a higher brain reserve,^42^, ^43^ which may partially explain exceptional cognitive performance in SAs.

In prior cross‐sectional studies, both our laboratory and others identified greater hippocampal volume in SAs.^8^, ^9^ However, when exploring longitudinal changes, contrasting results emerged.^12^, ^13^ In this study, we observed that hippocampal atrophy did not vary significantly based on CAG in models that also accounted for AD pathology. Instead, higher EC tau levels predicted faster hippocampal atrophy over time. Similarly, there was no evidence supporting a faster tau accumulation rate in EC and IT in individuals with an older cognitive age. Tau has been associated with regional MTL atrophy in cognitively normal older adults,^44^, ^45^ indicating a potential link between the pattern of hippocampal atrophy and tau accumulation in our study. On the other hand, we found no evidence that measures of tau or Aβ pathology predicted MCC atrophy, which was instead associated with CAG scores. These results may indicate a dissociation between AD‐related pathological features and other mechanisms associated with the preservation of MCC structural integrity over time. This interpretation finds further support in our longitudinal cognitive findings, where both CAG and AD pathology significantly predicted cognitive trajectories over time. Notably, the maintenance of the MCC integrity over time was associated with a slower multi‐domain cognitive decline, beyond the effects of Aβ and tau pathology. Conversely, EM decline was primarily linked to EC tau accumulation, indicating that MCC structural integrity may reflect broader aspects of cognitive functions, rather than AD‐related specific processes that preferentially affect EM. This observation aligns with the characterization of the MCC ROI itself, which arose from its association with the CAG, a multi‐domain measure of cognitive aging. Previous cross‐sectional investigations have reported lower levels of tau in SAs in the EC and IT.^4^, ^9^, ^46^ This suggests that SAs may result from a combination of processes, including protective factors like brain maintenance and reserve, particularly in the MCC, and resistance to AD and age‐related tau pathology. However, we found no differences in longitudinal tau, suggesting that once tau accumulation begins, participants with a younger cognitive age do not exhibit slower accumulation rates.

This study has some limitations. First, it is important to acknowledge that the BACS cohort is racially, ethnically, and socioeconomically homogeneous and highly educated, which does not reflect the full range of diversity in cognitive aging. Then, our definition of the cognitive continuum relies on a cross‐sectional measure. Features associated with exceptional cognitive performance may differ from those underlying cognitive maintenance over time. However, obtaining extensive longitudinal data to assess cognitive maintenance is challenging. Here we further validate our approach by showing that lower CAG scores were associated with slower cognitive decline over time.

In this study, we demonstrated that a younger cognitive age not only indicates exceptional cognitive performance but it is also associated with cognitive maintenance and slower atrophy progression in the MCC. These findings were robust when including measures of Aβ and tau in the models, suggesting that these features extend beyond the effects of AD pathology. Conversely, no differences were detected in longitudinal hippocampal atrophy and tau accumulation rates in relation to our measure of cognitive age. Taken together, these results suggest that distinct mechanisms may underlie protective MCC‐related processes compared to early AD and age‐related pathological changes. The combination of both mechanisms may contribute to exceptional performance and cognitive maintenance at an older age.

## AUTHOR CONTRIBUTIONS

Stefania Pezzoli and William J. Jagust conceptualized and designed the study. Stefania Pezzoli, Joseph Giorgio, Xi Chen, and Theresa M. Harrison contributed to the methodology and data analysis. Stefania Pezzoli and William J. Jagust wrote the manuscript and all authors contributed to the reviewing and editing of the manuscript.

## CONFLICT OF INTEREST STATEMENT

W.J.J. has served as a consultant to Lilly, Eisai, and Biogen. Author disclosures are available in the Supporting Information.

## CONSENT STATEMENT

All participants provided written, informed consent for their participation in this study.

## Supporting information



Supporting Information

Supporting Information
