# Supplementary material for: Cognitive aging outcomes are related to both tau pathology and maintenance of cingulate cortex structure
Source: Alzheimers Dement. 2025 Jan 14;21(2):e14515. doi: 10.1002/alz.14515 (PMC11848174; doi:10.1002/alz.14515)
Supplement: Supplementary file 1 — Supporting Information [file ALZ-21-e14515-s002.docx]

**Cognitive aging outcomes are related to both tau pathology and maintenance of cingulate cortex structure**

Stefania Pezzoli^1,2^, Joseph Giorgio^1,3^, Xi Chen^1,2,4^, Tyler J. Ward^1^, Theresa M. Harrison^1^, and William J. Jagust^1,2^

*^1^ Department of Neuroscience, University of California, Berkeley California, USA*

*^2^ Molecular Biophysics and Integrated Bioimaging, Lawrence Berkeley National Laboratory, Berkeley, California, USA*

*^3^ School of Psychological Sciences, College of Engineering Science and the Environment, University of Newcastle, Newcastle, New South Wales, Australia*

*^4^ Department of Psychology, Stony Brook University, Stony Brook, New York, USA*

**Supplementary materials**

**Supplementary tables**

**Supplementary Table S1** Subsamples cohort characteristics.

| Characteristic | MRI/PiB subsample (n = 92) | FTP subsample (n = 72) |
| --- | --- | --- |
| Age | 77.64 (4.84) | 77.14 (4.80) |
| Sex, female, n (%) | 53 (58) | 42 (58) |
| Education, years | 17.07 (1.84) | 16.96 (1.80) |
| History of hypertension, Y n (%) | 33 (36) | 23 (32) |
| Family history of dementia, Y n (%) | 31 (34) | 26 (37) |
| APOE ε4 n (%) | 26 (29) | 21 (30) |
| PiB+, n (%) | 44 (48) | 36 (50) |
| PiB Centiloids | 24.63 (32.87) | 25.24 (32.93) |
| CAG, years | 0.08 (4.64) | -0.58 (4.63) |
| MMSE | 28.61 (1.27) | 28.64 (1.27) |
| GDS ^a^ | 3.40 (3.13) | 3.28 (3.07) |

FTP: ^18^F-Flortaucipir; GDS: Geriatric Depression Scale; MMSE: Mini-Mental State Examination; MRI: magnetic resonance imaging; PiB: ^11^C-Pittsburgh compound B. Values represent Mean (SD) and N (%) for continuous and categorical variables respectively. ^a^ missing data for one participant.

**Supplementary Table S2** Predicting longitudinal change in cognition: linear mixed-effects models parameter estimates.

| **Parameter** | **Memory** | | | **Non-memory** | | | **Multi-domain cognition** | | |
| --- | --- | --- | --- | --- | --- | --- | --- | --- | --- |
|  | **Estimate** | **se** | ***p*-value** | **Estimate** | **se** | ***p*-value** | **Estimate** | **se** | ***p*-value** |
| **Intercept** | 130.77 | 3.45 | <0.001 | 57.91 | 3.52 | <0.001 | -0.12 | 0.13 | 0.36 |
| **Time** | -1.12 | 0.33 | 0.001 | -1.96 | 0.43 | <0.001 | -0.08 | 0.01 | <0.001 |
| **CAG** | -2.16 | 0.57 | <0.001 | -2.70 | 0.58 | <0.001 | -0.09 | 0.02 | <0.001 |
| **IT FTP SUVR** | -44.96 | 17.75 | 0.01 | -12.70 | 18.17 | 0.49 | -1.86 | 0.66 | 0.01 |
| **PiB DVR** | 6.17 | 13.08 | 0.64 | -5.49 | 13.32 | 0.68 | -0.28 | 0.49 | 0.56 |
| **Sex** | -0.72 | 5.33 | 0.89 | -10.48 | 5.44 | 0.06 | -0.43 | 0.20 | 0.03 |
| **Education** | 5.75 | 1.44 | 0.0001 | 5.13 | 1.47 | 0.001 | 0.21 | 0.05 | <0.001 |
| **CAG x time** | -0.12 | 0.06 | 0.046 | -0.03 | 0.08 | 0.73 | -0.003 | 0.002 | 0.18 |
| **IT FTP x time** | -5.95 | 1.90 | 0.003 | -1.36 | 2.44 | 0.58 | -0.33 | 0.07 | <0.001 |
| **PiB DVR x time** | -1.16 | 1.30 | 0.38 | -4.06 | 1.68 | 0.02 | -0.03 | 0.05 | 0.55 |
| **Sex x time** | 0.55 | 0.52 | 0.29 | 0.01 | 0.68 | 0.99 | 0.02 | 0.02 | 0.36 |
| **Education x time** | 0.22 | 0.14 | 0.13 | 0.35 | 0.19 | 0.07 | 0.01 | 0.01 | 0.02 |

CAG: cognitive age gap; DVR: distribution volume ratio; IT: inferior temporal; FTP: ^18^F-Flortaucipir; PiB: ^11^C-Pittsburgh compound B; ROI: region of interest; SUVR: standardized uptake value ratio.

**Supplementary Table S3** Predicting longitudinal atrophy: linear mixed-effects models parameter estimates.

| **Parameter** | **Midcingulate SA ROI** | | | **Hippocampus** | | |
| --- | --- | --- | --- | --- | --- | --- |
|  | **Estimate** | **se** | ***p*-value** | **Estimate** | **se** | ***p*-value** |
| **Intercept** | 0.43 | 0.04 | <0.001 | 0.60 | 0.05 | <0.001 |
| **Time** | -0.004 | 0.002 | 0.01 | 0.001 | 0.003 | 0.83 |
| **CAG** | -0.0002 | 0.001 | 0.78 | 0.001 | 0.001 | 0.36 |
| **IT FTP SUVR** | -0.02 | 0.03 | 0.46 | -0.04 | 0.03 | 0.18 |
| **PiB DVR** | -0.01 | 0.02 | 0.77 | 0.03 | 0.02 | 0.25 |
| **Sex** | -0.01 | 0.01 | 0.15 | -0.02 | 0.01 | 0.08 |
| **Education** | 0.001 | 0.002 | 0.55 | -0.003 | 0.003 | 0.31 |
| **CAG x time** | -0.0001 | 0.00003 | 0.002 | -0.00003 | 0.0001 | 0.59 |
| **IT FTP x time** | 0.002 | 0.001 | 0.16 | -0.003 | 0.002 | 0.11 |
| **PiB DVR x time** | -0.001 | 0.001 | 0.10 | -0.001 | 0.001 | 0.27 |
| **Sex x time** | 0.001 | 0.0003 | 0.08 | 0.0001 | 0.001 | 0.89 |
| **Education x time** | -0.0002 | 0.0001 | 0.05 | 0.0001 | 0.0001 | 0.43 |

CAG: cognitive age gap; DVR: distribution volume ratio; FTP: ^18^F-Flortaucipir; IT: inferior temporal cortex; PiB: ^11^C-Pittsburgh compound B; ROI: region of interest; SA: successful aging; SUVR: standardized uptake value ratio.

**Supplementary Table S4** Predicting longitudinal change in tau and β-amyloid: linear mixed-effects models parameter estimates.

| **Parameter** | **EC FTP SUVR** | | | **IT FTP SUVR** | | | **PiB DVR** | | |
| --- | --- | --- | --- | --- | --- | --- | --- | --- | --- |
|  | **Estimate** | **se** | ***p*-value** | **Estimate** | **se** | ***p*-value** | **Estimate** | **se** | ***p*-value** |
| **Intercept** | 1.19 | 0.04 | <0.001 | 1.20 | 0.02 | <0.001 | 1.14 | 0.04 | <0.001 |
| **Time** | 0.03 | 0.01 | 0.002 | 0.02 | 0.01 | 0.001 | 0.01 | 0.004 | <0.001 |
| **CAG** | 0.002 | 0.01 | 0.75 | 0.002 | 0.003 | 0.51 | 0.004 | 0.01 | 0.54 |
| **PiB DVR** | 0.31 | 0.11 | 0.01 | 0.23 | 0.06 | 0.001 | - | - | - |
| **Sex** | 0.02 | 0.05 | 0.75 | -0.02 | 0.03 | 0.59 | 0.07 | 0.05 | 0.18 |
| **Education** | -0.004 | 0.01 | 0.76 | <0.001 | 0.01 | 1.00 | -0.023 | 0.01 | 0.12 |
| **CAG x time** | -0.0003 | 0.001 | 0.76 | -0.001 | 0.001 | 0.34 | 0.0003 | 0.001 | 0.54 |
| **PiB DVR x time** | 0.04 | 0.03 | 0.12 | 0.05 | 0.02 | 0.02 | - | - | - |
| **Sex x time** | 0.001 | 0.01 | 0.91 | -0.008 | 0.01 | 0.38 | 0.002 | 0.005 | 0.69 |
| **Education x time** | -0.001 | 0.003 | 0.80 | 0.0002 | 0.003 | 0.94 | -0.002 | 0.001 | 0.17 |

CAG: cognitive age gap; DVR: distribution volume ratio; EC: entorhinal cortex; FTP: ^18^F-Flortaucipir; IT: inferior temporal cortex; PiB: ^11^C-Pittsburgh compound B; ROI: region of interest; SA: successful aging; SUVR: standardized uptake value ratio.

**Supplementary Table S5** Relationships between longitudinal change in cognition (cognitive composite slope), MCC GM slope, EC FTP slope and PiB DVR slope: multiple regression models parameter estimates.

| **Parameter** | **Memory slope** | | | **Non-memory slope** | | | **Multi-domain cognition slope** | | |
| --- | --- | --- | --- | --- | --- | --- | --- | --- | --- |
|  | **Estimate (se)** | **Standardized estimate** | ***p*-value** | **Estimate (se)** | **Standardized estimate** | ***p*-value** | **Estimate (se)** | **Standardized estimate** | ***p*-value** |
| **Intercept** | 0.50 (2.60) | - | 0.85 | 0.45 (4.91) | - | 0.93 | -0.07 (0.10) | - | 0.49 |
| **MCC GM slope** | 183.85 (252.42) | 0.09 | 0.47 | 725.81 (475.65) | 0.18 | 0.13 | 23.83 (9.95) | 0.27 | 0.02 |
| **EC FTP slope** | -27.47 (13.22) | -0.26 | 0.04 | -33.44 (24.91) | -0.17 | 0.18 | -0.72 (0.52) | -0.16 | 0.17 |
| **PiB DVR slope** | -16.62 (9.02) | -0.22 | 0.07 | -47.16 (16.99) | -0.32 | 0.01 | -0.82 (0.36) | -0.25 | 0.02 |
| **Age** | -0.02 (0.03) | -0.06 | 0.60 | -0.04 (0.06) | -0.08 | 0.48 | -0.001 (0.001) | -0.11 | 0.35 |
| **Sex** | 0.14 (0.28) | 0.06 | 0.61 | 0.81 (0.53) | 0.17 | 0.14 | 0.02 (0.01) | 0.24 | 0.03 |
| **Education** | 0.08 (0.08) | 0.11 | 0.34 | 0.19 (0.15) | 0.14 | 0.21 | 0.01 (0.003) | 0.30 | 0.01 |

DVR: distribution volume ratio; EC: entorhinal cortex; FTP: ^18^F-Flortaucipir; GM: gray matter; MCC: midcingulate cortex; PiB: ^11^C-Pittsburgh compound B.

**Supplementary figures**

**
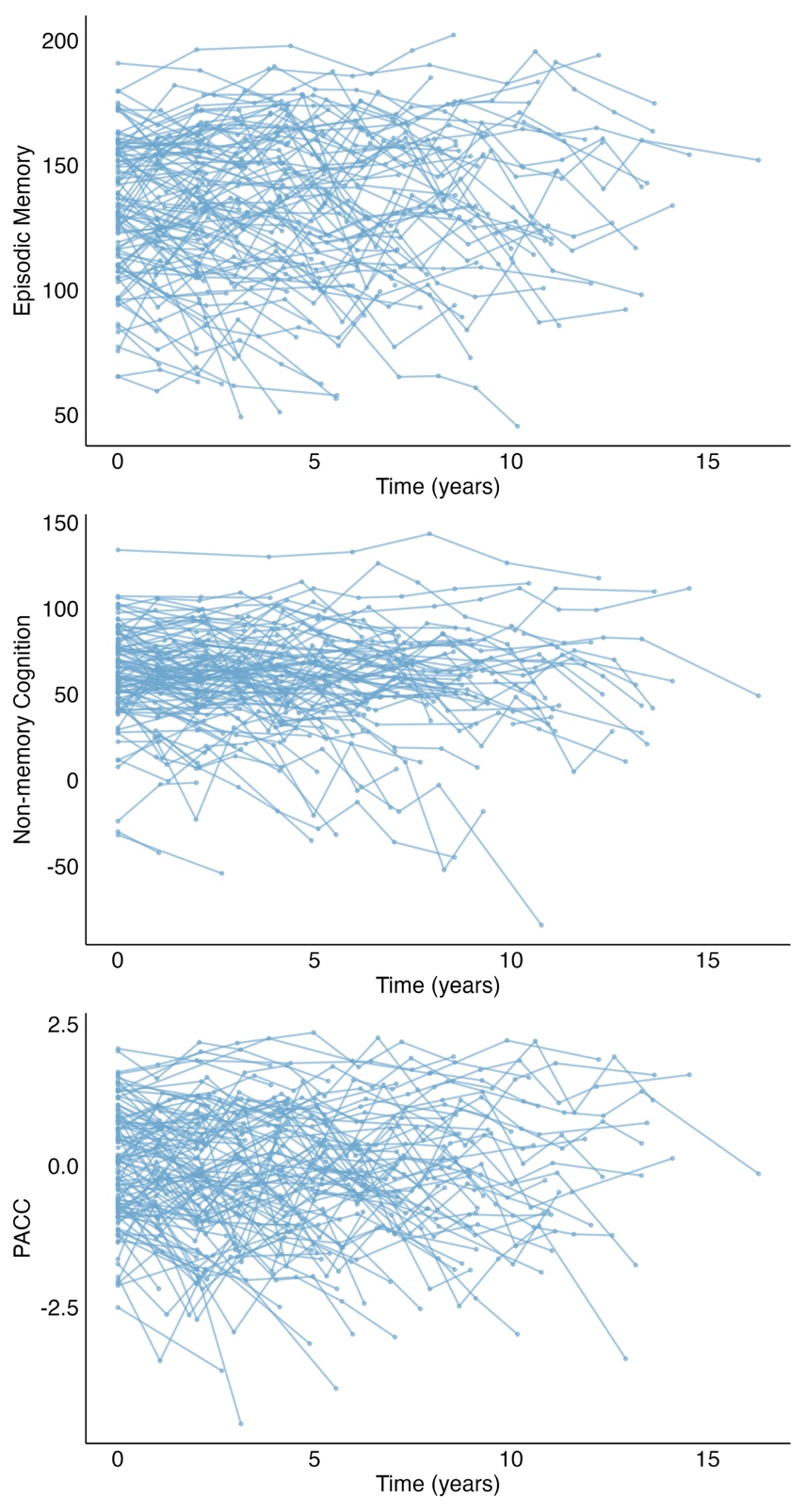
**

**Supplementary Figure S1** Spaghetti plots of cognitive scores over time across subjects, showing individual trajectories for Episodic Memory, Non-memory Cognition, and PACC scores. PACC: Preclinical Alzheimer Cognitive Composite.


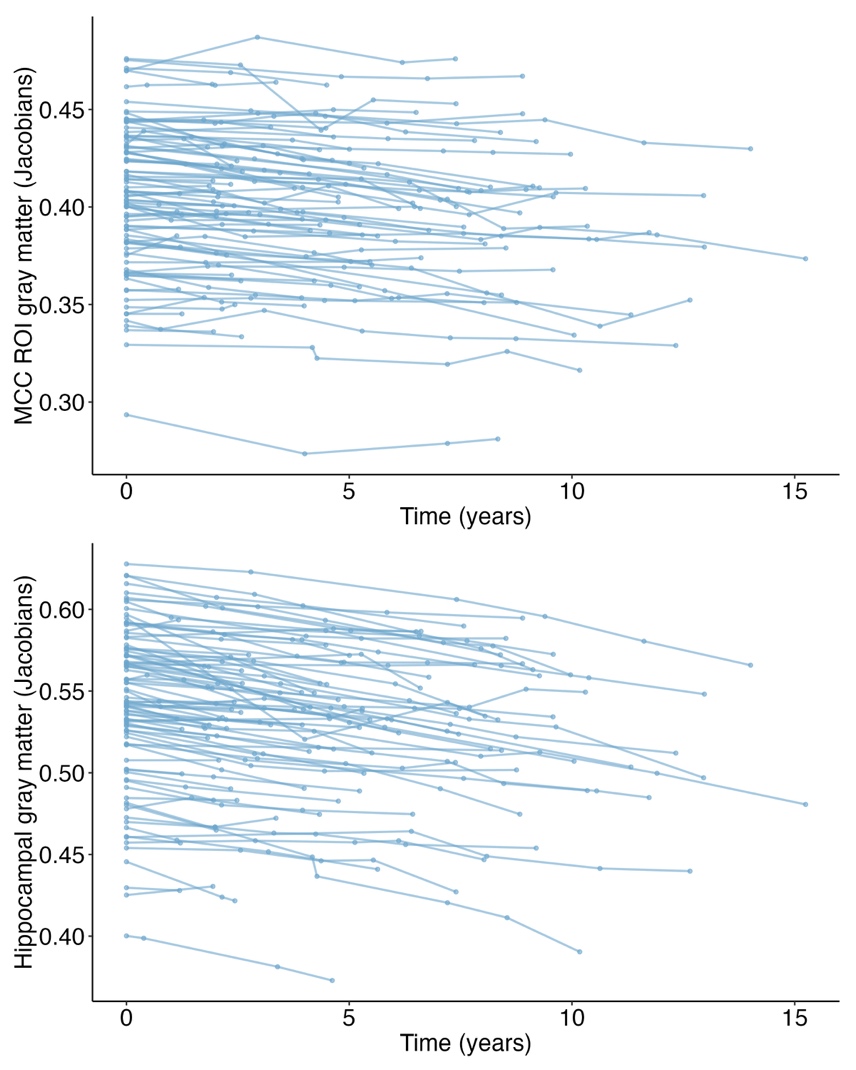


**Supplementary Figure S2** Spaghetti plots of gray matter Jacobian values over time across subjects, showing individual trajectories for MCC ROI, and hippocampus. MCC: midcingulate cortex; ROI: region of interest.
